# Does the smell of alcohol make it harder to resist? The impact of olfactory cues on inhibitory control and attentional bias

**DOI:** 10.1007/s00213-022-06073-0

**Published:** 2022-05-26

**Authors:** R. L. Monk, A. Qureshi, G. Wernham, D. Heim

**Affiliations:** 1 Edge Hill University, Saint Helens Road, Ormskirk, L39 4QP UK; 2 Liverpool Centre for Alcohol Research, Liverpool, UK

**Keywords:** Smell, Olfaction, Alcohol, Inhibition, Attention, Stroop, Go/no-go

## Abstract

**Background:**

It is well known that, owing to associative processing, olfactory cues can impact memory, emotion and behaviour. Research also points to a link between the smells of particular substances and craving. Yet, to date, little research has investigated how smell may impact other cognitive processes that are known to drive alcohol consumption.

**Aim:**

To assess how exposure to alcohol-related (vodka) relative to neutral (citrus) olfactory cues impacts inhibitory control and attentional bias.

**Method:**

Participants took part in a go/no-go (Study 1) and Stroop task (Study 2) while wearing masks that were pre-treated with vodka or citrus oil of equivalent intensity.

**Study 1 results:**

Response error rates were higher in participants in the alcohol-related (versus neutral) olfactory condition, with no interaction between olfactory and visual cue.

**Study 2 results:**

Responses to alcohol-related versus neutral words were similar, while performance appeared significantly impaired among participants wearing alcohol (relative to citrus) infused masks. Conclusion

The smell of alcohol may impair signal detection performance on the go/no-go and Stroop task. As inhibitory control and attentional processes are known to be associated with decisions to drink or exercise restraint, these results may have implications for our understanding of alcohol consumption and for tailoring interventions.

**Supplementary Information:**

The online version contains supplementary material available at 10.1007/s00213-022-06073-0.

Capitalising on human’s highly evolved and sensitive olfactory capabilities (McGann 2017), smells are harnessed to impact the behaviours of those exposed to them. Olfactory cues of a loved one, in this way, may improve sleep (Hofer and Chen 2020) and particular smells are released intentionally both inside and outside shops to nudge consumers into purchasing products (Jellinek 1997; Sandell 2019) by tapping into conditioned pleasant associations. Evidence further shows that smells have the potential to impact mood (Gottfried 2010; Herz 2009; Lehrner et al. 2005), memory (Gottfried 2010), task reaction times (Moss et al. 2003), anxiety (Lehrner et al. 2005; McCaffrey et al. 2009), cognitive performance (Moss et al. 2008) and pain perception (Gedney et al. 2004; Kim et al. 2006). Olfaction has also been found to impact the amount of effort exerted in a task (Herz et al. 2004) and interpersonal preferences (Li et al. 2007). Specifically, participants subjected to negative mood induction in the presence of an unfamiliar smell subsequently appear to spend less time on a task where the same unfamiliar smell is present, suggesting associative processing (Herz et al. 2004). The ability of olfaction to shape associated thoughts and behaviours is therefore well established within the literature.

The smell of alcohol can also impact the behaviour and thoughts of individuals. The scents of particular beverages may become associated with (un)happy memories, experiences or physiological responses, and it has been suggested that this drives subsequent approach or avoidance behaviours (Carter and Tiffany 1999). Indeed, through socialisation, even young children are able to recognise the smell of alcohol (Fossey 1993), especially when their parents drink heavily (Noll et al. 1990), and children also indicate preferences for particular types of alcoholic odours (Mennella and Garcia 2000). Evidencing further the pervasive associative properties of alcohol, laboratory research has demonstrated that it is possible to induce craving in participants by exposing them to alcohol-related smells (Litt and Cooney 1999) and, as such, asking participants to pick up and smell alcohol is an integral part of cue-induction paradigms, referred to as alcohol cue-elicited craving (e.g. Carter and Tiffany 1999; Kambouropoulos and Staiger 2001; Koukounas et al. 2019). The smell of alcohol has also been shown to increase self-reported desire to drink (Laberg 1990). Somewhat relatedly, exposure to food smells has also been shown to increase eating in overweight populations (Fedoroff et al. 2003; Ferriday and Brunstrom 2011; Jansen and van den Hout 1991; Jansen et al. 2003), while work has found that pleasant olfactory cues may reduce smoking-related (Sayette et al. 2019) and food-related (Kemps and Tiggemann 2013) craving. A body of work therefore suggests that smells may shape craving responses for appetitive stimuli and, given that craving is just one of a number of processes implicated in alcohol consumption (e.g. see McNeill et al. 2021), it remains to be questioned whether olfactory cues may also exert influences over other cognitive processes implicated in driving alcohol behaviours.

Neurological research affords insights into how alcohol smells may impact those who sense them. For example, exposure to alcohol-related olfactory cues (e.g. whisky, in contrast to neutral cues such as grass or leather) has been shown to be associated with increased activation in the nucleus accumbens and ventral tegmental areas — regions of the brain associated with reward anticipation in heavy drinkers (Kareken et al. 2004). The smell of alcohol therefore appears to trigger associated alcohol-related beliefs, expectations and cognitive processes which, in turn, may drive consumption. The dopaminergic system, including the nucleus accumbens and the ventral striatum, has also been associated with inhibitory control (Pattij et al. 2007), reward-related behaviour that is associated with ingestion (e.g. Kelley 2004) and problem consumption (Volkow et al. 2009). Consequently, there is reason to believe that alcohol-related smells may be associated with activation in areas of the brain that are implicated in inhibitory control (the ability to control impulsive responses) and the regulation of ingestive behaviours. This growing area of research offers insights into the olfactory-driven associative processes that may drive (or indeed diminish) consumption.

The assertion that alcohol-related cues may impact cognitive processes implicated in alcohol consumption appears supported by initial research by Monk et al. (2016) who, heeding a call for the impact of olfaction on inhibitory control to be elucidated (Schacht et al. 2013), found that alcohol-related smells appeared to weaken inhibitory control as measured by the go/no-go task. These findings correspond to research indicating that (non-olfactory) cues elicit a psychomotor-activating response (Wiers et al. 2002), leading to difficulties in inhibiting a dominant response (Roberts et al. 2014). It also extended prior research on other sensory modalities, where alcohol-related sights and sounds have been shown to weaken inhibitory control and be implicated in attentional biases, where priority of attention/focus is given to alcohol-related stimuli (Field and Jones 2017; Kreusch et al. 2013; Qureshi et al. 2018; Qureshi et al. 2018, 2019; Monk et al. 2017; Pennington et al. 2019a; Pennington et al. 2019b; Sharma et al. 2001; though see Baines et al. 2019; Jones et al. 2018).

In summary, a growing body of research suggests that alcohol-related cues can induce craving, and that smells may also be harnessed to satiate desire for (non-alcoholic) substance consumption. It is also well established that visual and auditory sensory modalities can impact people’s inhibitory control and attention, through their associative properties, and that these cognitive processes may, in turn, shape consumption or restraint. However, whether the same is also true of olfactory cues has received scant research attention. Building on preliminary finding from Monk et al. (2016), the aim of this study was therefore to examine whether alcohol-related olfactory cues impact inhibitory control and attentional bias, in a similar fashion to the effects observed for alcohol-related sights and sounds. It was predicted that performance on the go/no-go task and the Stroop would be impaired in response to alcohol-related images in the go/no-go task and (versus neutral) words in the Alcohol Stroop tasks. In accordance with the findings from Monk et al. (2016), it was also hypothesised that participants would perform worse on both tasks when taking part in the presence of alcohol-related olfactory cues (vodka versus neutral/citrus). In short, we expected inhibitory control to be lower and attentional bias to be higher when participants could smell alcohol-related odours.

## Analytic approach and justification

Participant performance for both studies was calculated through Signal Detection Theory (SDT; Ben-David et al. 2012; Macmillan and Creelman 1991). Detection theory is an approach to measuring decisions made under uncertainty, whereby the decision is affected by sensitivity to the physical characteristics defining the stimuli (hereby alcohol or non-alcohol related), and by the response bias regarding favouring one stimulus over another when responding. This approach allows us to assess whether changes in performance are due to sensory modification, indicated by a difference in sensitivity scores (*d′*), or in cognitive adjustment, shown by a difference in response bias (*β*).

SDT allows the calculation of a sensitivity index (*d′*) for the detection of a given signal, based on whether the signal is present or absent and if it is then detected or not. *D′* was calculated for neutral and alcohol stimuli based on accuracy rates for each olfactory group

(following Macmillan and Creelman 1991). The formula used for d prime was *D′ = z(H) − z(FA)*, where *z(H)* and *z(FA)* are the z transformations of the hit and false alarm rates, respectively (Z transforms calculated using the Excel function NORMSINV). As such, larger values of *d′* indicate that the signal is obviously different from the background or noise of the task. In other words, larger scores reflect better task performance. The calculation of *d′* scores allows the comparison of performance between studies with different methodological approaches. In Study 1, NoGo trials are in the minority of trials, while alcohol/neutral words are balanced in Stroop task. Therefore *d′* provides a mechanism for comparing the results of two studies (e.g. Jang et al. 2010). Analyses of reaction times, accuracy and interference are available in supplementary materials.

Response bias scores were also calculated using *β* =  *exp* [0.5 ∗ ((*z*(*FA*))2 − (*z*(*H*))2)]. This response bias score (*β*) gives the propensity of the participant to respond (or not), with an unbiased participant having values of around 1.0. As the bias to respond increases, resulting in higher hit rate and higher false alarm rate, this value will approach zero. As the bias to not respond increases, resulting in lower hit rate and false alarm rate, this value will increase to over 1.0. When analysing the effects of both *d′* and *β* scores, deficiencies in attention are indicated by differences in *β* scores in response to stimuli, while a temporary disruption of action can be derived from difference in *d′* scores between stimuli (Ben-David et al. 2012).

## Study 1

### Participants

Forty participants (53% female) aged 18–25 years old (M = 22.76 SD = 2.67) were recruited via responses to an advertisement seeking regular social drinkers (Mean AUDIT = 10.12 (7.05) 1) defined as those who regularly consume more than 2 drinks in a given drinking episode). Based on previous research examining olfactory cue and alcohol-related stimuli (Monk et al. 2016), the required sample size was calculated to be 36^2^ (G*Power; Faul et al. 2009).

Participants were randomly allocated to either alcohol (*n* = 20) or neutral olfactory cue conditions. Preliminary analyses suggested that there were no significant differences in the age, gender or AUDIT scores between these groups (*p* > .05; see Table 1).

**Table 1:** Demographic and drinking measure descriptives for Study 1

	M	F	AUDIT	Age
Vodka cue	8	12	9.14 (7.53)	23.21 (6.99)
Citrus cue	9	11	9.38 (6.44)	24.56 (7.88)

### Design

A 2 (Visual Stimuli: Alcohol and Neutral) x2 (Olfactory Cues: Alcohol or non-alcohol) mixed-groups design explored the effect of olfactory cues and visual stimuli on participants’ go/no-go task performance, assessed via *d′* scores and *β* scores calculated through signal detection theory (SDT; Ben-David et al. 2012; Macmillan and Creelman 1991), which allows the calculation of a sensitivity index (*d′*) for the detection of a given signal, based on whether the signal is present or absent and if it is then detected or not. Larger values of *d′* indicate that the signal is obviously different from the background or noise of the task. In other words, larger scores reflect better task performance. *β* scores of around 1.0 indicate no particular bias to responding or not responding. Values closer to 0 indicate a bias to respond, whereas values above 1.0 indicate a bias to not respond.

### *Stimuli and materials*

#### The alcohol use disorder identification test (AUDIT- Saunders et al. 1993)

AUDIT is a 10-item questionnaire which examines hazardous and harmful alcohol use. A score of 8 or more has been deemed hazardous or harmful alcohol use (Saunders et al. 1993). This measure showed good reliability (Cronbach’s *α* = .72).

#### Olfactory cues

Following the paradigm implemented by (Monk et al. 2016), a mask was worn by all participants. This was pre-treated using a pipette with small amounts of vodka (5 ml of diluted Eristoff vodka, 1:5 dilution, administered as the alcohol-related olfactory cue) or citrus oil (5 ml of diluted oil, 1:10 dilution, as the control condition) based on pilot testing to balance stimuli liking and to control odour concentrations (Smeets and Dijksterhuis 2014). A full outline is supplied in Monk et al. (2016).

#### The go/no-go association task

Based on a research by Kreusch et al. (2013) and Monk et al. (2016), the go/no-go association task used in this research utilised two picture sets for the visual cues: one set contained neutral pictures (the letter K vs. the other 25 letters) and the other set with bar-related pictures (a beer bottle vs. 25 water bottle pictures). Pictures of the letter K and beer were the target stimuli (14% were no-go; 36 no-go, 224 Go stimuli used). Go/no-go tasks use this low ratio of no-go/go trials to create a response prepotency that is difficult to inhibit on NoGo trials (Kaufman et al. 2003). Split-half reliability was calculated for each olfactory cue group, separately for go and no-go trials. All values were above .97. The E-Prime 2.0 software was used to design and implement this procedure.

### Procedure

This research was approved by the appropriate University ethics committee and performed in accordance with the ethical standards laid down in the 1964 Declaration of Helsinki. All persons gave their informed consent prior to their inclusion in the study.

Participants who signed up to take part were randomly assigned to one of two olfactory conditions. Upon arrival at the pre-arranged testing laboratory, they entered the room, sat in front of the provided computer and were given a study briefing, prior to signing their consent and completing basic demographic and AUDIT questionnaires. They were then asked to put on the mask and headphones (which they were informed were designed to limit the potential impact of external noise, light or smells on performance^3^) before following the on-screen task instructions for the completion of go/no-go paradigm.

Participants were required to inhibit their response to target stimuli (see materials; alcohol condition = bottle of beer; neutral condition = letter K), but to respond to all other stimuli. A feedback tone (250 ms) was delivered if participants responded incorrectly. The experiment was organised into 16 randomised blocks, eight with alcohol visual stimuli and eight with neutral (letter) stimuli. Trial order was pseudo-randomised so that no more than 3 of any given trial were permitted to be delivered in a row and no blocks could start with a no-go trial).

The study lasted approximately 30 min and included breaks between blocks to mitigate fatigue. The mask was removed at the end of testing and participants were debriefed as to the true nature of the study, while being asked not to share this with other potential participants.

## Results

### Sensitivity index (d′)

*D′* scores were submitted to a 2 × 2 mixed ANOVA with between-participants conditions of olfactory group (alcohol smell × neutral smell) and a within-participants condition of stimuli type (alcohol stimuli × neutral stimuli).

**Fig. 1:**
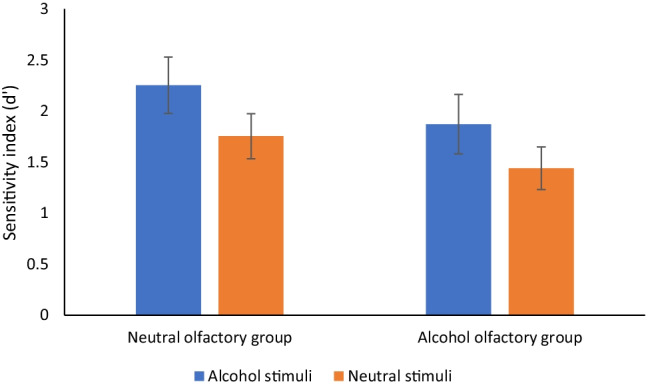
Sensitivity index (*d′*) by word type and olfactory group (bars = confidence intervals) for go/no-go

Results showed a main effect of stimuli type with higher *d′* scores for alcohol pictorial targets compared to neutral (letters) pictorial targets (*F* (1, 38) = 30.76, *p* < .01, η_p_^2^ = .45). There was also a main effect of olfactory group, with higher *d′* scores for the neutral group compared to the alcohol group (*F* (1, 38) = 4.66, *p* < .05, η_p_^2^ = .11) There was no interaction between stimuli and group *(F* (1, 38) = .17, *p* = .66, η_p_^2^ = .00). These results are displayed in Fig. 1. This suggests that while neutral pictorial targets were more difficult to detect than alcohol pictorial targets, the alcohol olfactory group found it harder to detect pictorial targets in general, compared to the neutral olfactory group.

### Relationship with AUDIT scores

In order to test potential associations between AUDIT scores and magnitude of inhibitory control, we also conducted a series of correlations.

Correlations between AUDIT scores and Go response times, Go trial accuracy, false alarm rates and *d′* scores (for alcohol and neutral stimuli) for each olfactory cue group were analysed for Study 1. There were significant correlations between AUDIT and alcohol stimuli Go RT (*r* = .47, *p* = .04) and neutral stimuli Go RT (*r* = .48, *p* = .03) for the alcohol olfactory cue group, with no other significant correlations in that group and none for the neutral olfactory cue group.

### Response bias (β)

**Fig. 2:**
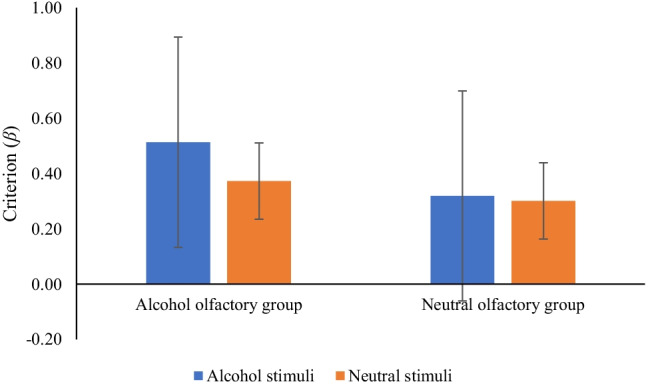
Response bias (*β*) by word type and olfactory group (bars = confidence intervals)

*β* scores were submitted to a 2 × 2 mixed ANOVA with between-participants conditions of olfactory group (alcohol smell × neutral smell) and a within-participants condition of stimuli type (alcohol stimuli × neutral stimuli). Results showed no main effect of word type (*F* (1, 38) = .64, *p* = .43, η_p_^2^ = .02) or olfactory group (*F* (1, 38) = .59, *p* = .45, η_p_^2^ = .02). There was no interaction between word type and olfactory group (*F* (1, 38) = .38, *p* = .54, η_p_^2^ = .01). These results are displayed in Fig. 2.

## Study 2

### Participants

Forty participants (50% female), aged 18–25 years old (M = 20.87 SD = 1.22) who reported regularly consuming more than two alcoholic beverages in one instance^4^, were recruited for this study via opportunity sampling in response to a research advertisement. Power analyses were carried out using G*Power, as outlined in Study 1. Preliminary analyses suggested that there were no significant differences in the age or AUDIT scores between these groups (*p* > .05; see Table 2), though there was a difference in gender mix (Χ^2^ (1, *N* = 40) = 5.01, *p* = .03).

**Table 2:** Demographic and drinking measure descriptives for Study 2

	M	F	AUDIT	Age
Vodka cue	5	15	16.6 (6.58)	20.87 (1.29)
Citrus cue	12	8	17.1 (6.90)	20.87 (1.12)

### Design

The design was a 2 (Olfactory cues: Vodka or Citrus) × 2 (Word type: Alcohol and neutral) mixed group design in order to investigate the effects of olfactory and visual cues on *d′* in the Alcohol Stroop Task (e.g. Ben-David et al. 2012).

### Stimuli and materials

The alcohol use disorders identification test (Cronbach’s α = .85) and olfactory stimuli were the same as those used for Study 1.

#### The alcohol Stroop task (Bauer and Cox 1998)

The current study used an adapted version on Inquisit. Participants were instructed to respond as quickly as possible to the colour of the word presented (ignoring the meaning of the word) by pressing keys that were affixed with corresponding coloured stickers. Each word was presented in red, yellow, blue or green on a white background. Trials consisted of an initial fixation cross for 500 ms, followed by the stimulus word. The stimulus remained on-screen for 1500 ms or until the participant responded, followed by a 200-ms inter-trial interval. The experiment started with 24 practice trials consisting of numbers (from one to ten) presented in one of the four colours. Following these, two blocks of 160 trials using a mixture of alcohol-related (e.g. vodka) and neutral words (e.g. sweater) were presented, with a 2-min rest between blocks to prevent fatigue. Both the alcohol-related and neutral stimulus categories consisted of 15 stimuli, which were presented between 5 and 6 times in each block, and with the order of stimuli and colour, they were presented in randomised order for each participant. The slowing effect usually seen when the semantic content of the word is relevant to the individual’s concern is termed the Stroop or interference effect and is suggested to be due to an attentional bias for the concern-related information of certain words, this case being alcohol-related (Bruce and Jones 2004). Split-half reliability was calculated for each olfactory cue group separately for neutral and alcohol words. Values for the neutral olfactory cue group was .63 for neutral words and .90 for alcohol words, while for the alcohol olfactory cue group, the respective values were .60 for neutral words and .85 for alcohol words.

### Procedure

The testing environment, cover story, briefing and debriefing procedures were the same as in Study 1, as was the olfactory cueing protocol. The computer-based task completed in this study was the Alcohol Stroop (rather than the go/no-go task) and testing took approximately 30 min in total.

## Results

### Sensitivity index (d′)

*D′*, a sensitivity index, was calculated for neutral and alcohol words for each group, based on accuracy rates (as per Ben-David et al. 2012). These were used in a 2 × 2 mixed ANOVA with between-participants conditions of olfactory group (alcohol smell × neutral smell) and a within-participants condition of word type (alcohol word × neutral word).

**Fig. 3:**
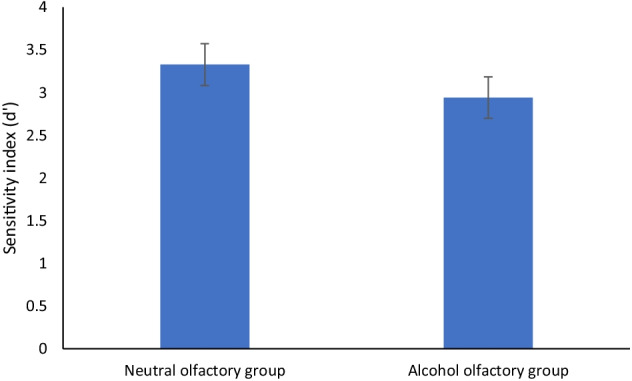
Sensitivity index (*d*′) by olfactory group (bars = confidence intervals) for Stroop

Results showed no main effect of word type (*F* (1, 38) = .04, *p* = .85, η_p_^2^ = .00), but a main effect of olfactory group (*F* (1, 38) = 5.17, *p* < .05, η_p_^2^ = .12), with higher *d′* scores for the neutral olfactory group. There was no interaction between word type and olfactory group (*F* (1, 38) = 3.79, *p* = .06, η_p_^2^ = .09) demonstrated in Fig. 3.

### Relationship with AUDIT scores

In order to test for potential associations between AUDIT scores and magnitude of attentional bias, a series of correlations. Correlations were carried out between AUDIT and alcohol word latency, neutral word latency, alcohol interference effect and *d′* scores for alcohol and neutral words for both olfactory cue groups in Study 2. No significant correlations were found (all *p*’s > .20).

### Response bias (β)

**Fig. 4:**
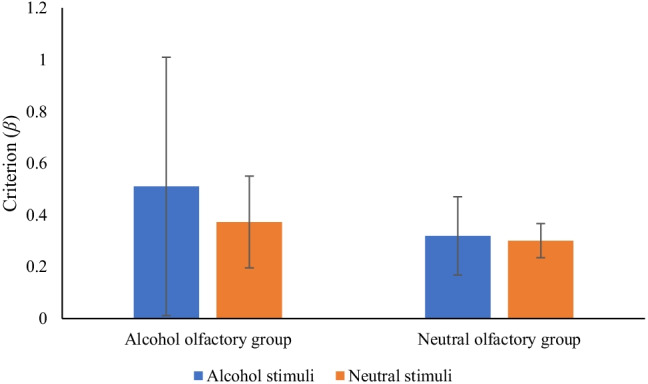
Response bias (*β*) by word type and olfactory group (bars = confidence intervals)

*β* scores were submitted to a 2 × 2 mixed ANOVA with between-participants conditions of olfactory group (alcohol smell × neutral smell) and a within-participants condition of stimuli type (alcohol stimuli × neutral stimuli). Results showed no main effect of word type (*F* (1, 38) = .62, *p* = .44, η_p_^2^ = .02) or olfactory group (*F* (1, 38) = .58, *p* = .45, η_p_^2^ = .02). There was no interaction between word type and olfactory group (*F* (1, 38) = .37, *p* = .55, η_p_^2^ = .01). These results are summarised in Fig. 4.

## Discussion

Building on research pointing towards the potential of olfactory cues to impact a range of behaviours and responses, the aim of this study was to examine whether alcohol-related smells impact inhibitory control and attentional bias in a similar fashion to the effects observed for visual and auditory sensory cues. In accordance with hypotheses, signal detection performance on the go/no-go task was impaired when participants were exposed to the smell of vodka (versus citrus). These findings support earlier research by Monk et al. (2016) and build upon neurological imaging research which suggests that alcohol-smells are associated with activation of areas of the brain (e.g. nucleus accumbens and the ventral tegmental areas; Kareken et al. 2004) that are associated with inhibitory control (Pattij et al. 2007). The present results consequently add credence to the suggestion that alcohol-related smells impair signal detection on tasks which require inhibitory control, potentially via associative processes which link (the smell of) alcohol with previous consumption experiences. Furthermore, that analyses suggested that there was a relationship between AUDIT scores and the magnitude of Go Trial responses in the olfactory condition (in terms of reaction times) may also suggest that those with more problem drinking may be particularly susceptible to response activation during olfactory cue exposure. The current findings therefore indicate that alcohol-related smells may reduce people’s general ability to control prepotent responses to appetitive visual cues, and that those with higher levels of problem drinking may be more susceptible to this.

The results of Study 2 were also in-line with predictions and offer novel insights into the impact of olfactory cues on attentional bias. Here, signal detection on the Stoop task appeared to be significantly impaired among participants wearing alcohol (relative to citrus-)-infused masks, irrespective of the nature of the visual stimuli. Extending findings suggesting that alcohol-related smells may elicit craving (e.g. Litt and Cooney 1999) and impair inhibitory control (Monk et al. 2016), the current work therefore suggest that associative (conditional) processes activated by olfactory cues also impact attention such that signal detection is affected. Indeed, our findings appear to align with the notion that (non-olfactory) cues elicit a psychomotor-activating response (Wiers et al. 2002), associated with difficulties in inhibiting responses, and extend to olfaction findings from previous work which has hitherto largely focused on the attentional and inhibitory impacts of auditory and visual sensory modalities. Finally, given that *d′* provides a mechanism for comparing the results of studies with varying methodologies and numbers (incorporate hit/false alarms), the current findings may also suggest that alcohol-related cues may exert a stronger impact on signal detection performance as assessed by the go no-go versus the Stroop task (as *d′* was higher in the Stroop). Further analysis in this regard is, nevertheless, recommended to test this tentative assertion.

The results from response bias analyses also offer further evidence for the effect of alcohol-related smells on responses. Here, following the approach of Ben-David et al. (2012), response bias analyses (*β*) were carried out. In their study, signal detection was used to determine whether the emotional Stroop effect was due to deficient attention to colour (apparent by differences in *β*) or because of temporary disruption of action in the face of threat (evidenced by differences in *d′*: ibid). The current results were analogous to those of Ben-David and colleagues (i.e. there was an effect on *d′* but not on *β* scores), although we found an effect on effect on olfactory cue (alcohol × citrus) as opposed to word type (emotional/threatening × neutral). According to Ben-David et al. (2012), such results (in our case, slower/worse detection in the alcohol olfactory group) are due to an instinctive perceptual-motor reaction to the stimuli, rather than changes in criterion to responding/stimuli content. As such, the current findings provide evidence that there may be an instinctual perceptual-motor reaction to the smell of alcohol (impacting both inhibitory control and attentional bias) rather than a deficiency in attention to the smell of alcohol.

While results relating to the effect of olfactory cues were as expected, it should be noted that there were a number of unanticipated findings surrounding (non) alcohol-related visual stimuli. First, Study 1 inhibitory control performance was better in response to alcohol than non-alcohol visual stimuli, in contrast with other findings which suggests impaired inhibition to alcohol-related stimuli (e.g. Weafer and Fillmore 2012). One explanation for this observation may be that variety of Go stimuli was much higher for non-alcohol stimuli. Specifically, for the non-alcohol stimuli there were 25 different letters to respond to (while ignoring the letter K), but for the alcoholic stimuli there was only 1 water bottle to respond to and 1 beer bottle image to ignore, which may have simplified the task demands. Furthermore, in Study 2, while responses to alcohol words were slower than to non-alcohol words (as outlined in supplementary materials), no differences in response performance were evident between alcohol-related visual cues meaning that attentional bias did not differ in response to alcoholic and neutral words. This finding is unexpected in light of previous research indicating a typical ‘alcohol Stroop’ whereby attentional bias is higher to alcohol-related words, affecting task performance (e.g. Bauer and Cox 1998; Hallgren and McCrady 2013; Johnsen et al. 1994), particularly among heavier drinkers, as is the case in the current study based on their AUDIT scores (e.g. Sharma et al. 2001). This observation would accord with suggestions that responses to alcohol-related visual cues may spill over (or generalise) to other appetitive cues (Monk et al. 2017 ; Pennington et al. 2019b). That this was not evident for Study 1, however, casts some doubt on this assertion and further research is advised, however, in order to test this assertion.

### Implications, limitations and conclusions

The current findings are important for two reasons. First, findings from research carried out in olfactorily neutral laboratories (i.e. in the absence of alcohol-related smells) may not fully generalise to real world environmental contexts (such as bars), where such alcohol cues are ubiquitous. Second, interventions seeking to target alcohol-related behaviours may benefit from accounting for olfactory cues to shape behavioural responses, given that inhibitory control and attentional bias are well-established cognitive processes which impact alcohol consumption. For example, it may be prudent for individuals wishing to abstain from alcohol to avoid locations where the smell of alcohol is prevalent. These findings, in this way, represent a further step towards enhancing our knowledge of the effects of olfaction on alcohol-related inhibitory control and attentional bias. Nevertheless, we suggest that a non-odour condition be included in future explorations, as well as additional types of alcoholic cues. This will allow researchers to assess which types of alcohol (e.g. beer) elicit the greatest behavioural or cognitive responses (Schneider et al. 2001) and to guard against the possibility that the citrus smell may also exert an influence on participants’ responses (Smeets and Dijksterhuis 2014). Another reason for expanding the alcoholic beverages used in future research of this nature is that we did not record participants’ beverage preferences prior to their taking part. As such, the current research is unable to rule out individual variability in respondents’ (dis)likes of, or propensity to drink, vodka and recent research has highlighted the potential importance of such considerations (e.g. see work using the bogus/ad libitum taste test (Jones et al. 2016)). While matching beverage preferences to each individual within a single olfactory study may be difficult, owing to variances in smell intensity between alcoholic beverages (beer may be less intense than vodka, for example), future research is warranted to explore this possibility and assess the extent to which current findings replicate with other drinks.

For the purposes of the go/no-go task, we compared performance on letter stimuli (neutral) v pictorial stimuli (alcohol). While this approach in keeping with previous research (Monk et al. 2016) and neutral stimuli often are unrelated to experimental stimuli (e.g. stapler vs beer; Kreusch et al. 2013), we would however note that letters and pictures belong to different semantic categories, which may have affected responses. This ought to be born in mind when considering the current results. As context-related cueing may be particularly likely in the student-based sample (Rumelhart and Todd 1993) utilised in our research, we would also caution against generalising the current findings beyond this population. In addition, we note that the effect of impulsivity during testing and baseline variability in impulsiveness between participants cannot be ruled out as possible influences on current findings. Finally, in relation to the tasks selected for the current research, it should be remembered that the go/no-go task involves both response selection *and* inhibition, rather than just the latter. Likewise, it has been suggested that the Stroop task may also measure both selective attention (Logan 1980) and inhibition (Sugg and McDonald 1994), tapping into multiple underlying processes (see Jones et al. 2021). Future research could therefore fruitfully utilise measures such as the stop signal or the anti-saccade task to expand the methods used in this research domain.

Expanding the growing body of research which suggests that olfactory cues can assert powerful influences on people’s thoughts and behaviours, the current research built upon previous studies which suggest that alcohol-related smells impact craving. In doing so, it also addressed a relative lack of research with an explicit focus on inhibitory control and attention in this area. Findings suggest that, possibly owing to the associate processes it elicits, the smell of alcohol can impair signal detection performance on both go/no-go and Stroop tasks. The current study therefore extends formative work (Monk et al. 2016) and afforded novel insights into the potential for alcohol-related olfactory cues to tasks of inhibitory control and attention. Given that these cognitive processes are implicated in alcohol consumption behaviours, findings may have implications for the design of research environments which more realistically mimic real-world contexts. As such, alcohol-related smells may warrant closer attention as a potential modifiable influence on the cognitive and behavioural responses under investigation in laboratory settings. Intervention efforts to reduce heavy drinking may also benefit from awareness of the potential associative power of alcohol-related smells to elicit particular responses.

## Supplementary Information

ESM 1 (DOCX 36 kb)

## Footnotes

1 While slightly above the cut-off for clinical assessment (scores of 8 or above being deemed to indicate hazardous or harmful alcohol use) (Babor, de la Fuente, Juan Ramón, Saunders, & Grant, 1992 ; Saunders et al., 1993), participants are comparable with recent research using UK student samples (Clarke, Field, & Rose, 2015; Monk et al. 2016; Moss et al., 2015)

2 *α* = .05, observed power = .8, *f* = .39

3 Post-test manipulation tests suggested that while participants did notice a slight smell in the mask, they did not infer the aims of the study not suspect that olfaction was a key study variable.

4 This approach of attempting to more specifically define social drinking mirrors similar published approaches (Monk et al. 2021; 2020; 2017)
