# Supplementary material for: Does the smell of alcohol make it harder to resist? The impact of olfactory cues on inhibitory control and attentional bias
Source: Psychopharmacology (Berl). 2022 May 26;239(7):2109–18. doi: 10.1007/s00213-022-06073-0 (PMC9205803; doi:10.1007/s00213-022-06073-0)
Supplement: Supplementary file 1 — (DOCX 36 kb) [file 213_2022_6073_MOESM1_ESM.docx]

**Supplementary materials file 1**

**Alternative Task Measures**

Study 1 (GNG)

Three analyses were conducted: one with FAR as the dependent variable, one with reaction times on Go trials (ms), and one with response accuracy on Go trials[[1]](#footnote-1). Means and standard deviations of participants' FAR, accuracy and reaction times across olfactory and visual cueing conditions are reported in Table 2. A main effect of olfactory cue was found (*F*(1, 38) = 5.42, *p* =.03, $\eta_{p}^{2}$= .13) such that the FAR was higher for those receiving the alcohol olfactory cue (M = .56, SD = .04) than for the control olfactory cue (*M* = .42, *SD* = .04). Furthermore, there was a main effect of pictorial target (*F*(1, 38) = 15.65, *p* ≤ .01, $\eta_{p}^{2}$ = .29), such that the FAR was higher for the neutral (letters) pictorial targets in comparison with the alcohol pictorial targets. There was no significant interaction between olfactory cue and pictorial target (*F*(1, 38) = .29, *p* = .60, $\eta_{p}^{2}$ = .01).

***Reaction time on Go Trials***

Whilst there was no main effect of olfactory cue on reaction time (*F*(1, 38) = .16, *p* > .69, $\eta_{p}^{2}$= .00), a significant main effect of pictorial target on reaction time was found (*F*(1, 38) = 4.77, *p* = .04, $\eta_{p}^{2}$= .11) such that reaction time was slower for the neutral pictorial target than for the alcohol pictorial target. There was no significant interaction between olfactory cue and the pictorial target (*F*(1, 38) = .93, *p* = .34, $\eta_{p}^{2}$= .02).

***Accuracy on Go Trials***

There was no main effect of olfactory cue on go accuracy rates (*F*(1, 38) = .76, *p* = .39, $\eta_{p}^{2}$= .02) with the accuracy slightly higher for the control olfactory cue than the alcohol olfactory cue. Yet there was a main effect of pictorial target on accuracy (*F*(1, 38) = 4.26, *p* = .05, $\eta_{p}^{2}$= .01), such that accuracy was significantly higher for the alcohol pictorial target than the neutral pictorial target. There was no significant interaction between olfactory cue and pictorial target (*F*(1, 38) = .71, *p* = .41, $\eta_{p}^{2}$= .02).

Study 2 (Stroop)

Mixed ANOVA with a within-subject word type (alcohol x neutral; latency, correct only) and a between-subjects factor cue type (vodka x control) shows a main effect of word type *(F* (1, 38) = 4.29, *p* = .045, η_p_^2^ = .10; slower responses to alcohol words) but no main effect of cue type (*F* (1,38) = .15, *p* = .70, η_p_^2^ = .00) or interaction between word and cue type (*F* (1, 38) = 3.99, *p* = .053, η_p_^2^ = .10).

Examining the interaction anyway shows that while there was no difference in response times between cue types for either word type (*p*’s > .49), participants cued with vodka had significantly longer response times to alcohol words (compared to neutral words; *p* = .007). Participants in the control cue condition showed no difference in response times between alcohol and neutral words (*p* = .96).

Running a straight between-subjects ANOVA (cue type: vodka x control) with an interference measure as the dependent variable (alcohol word – neutral word response time) showed a main effect of cue type (F (1, 38) = 5.58, *p* = .023, η_p_^2^ = .13). A larger interference effect was shown for those cued with vodka relative to the control cue group (*M* = 11.67, *SE* = 3.44 compared to *M* = .18, *SE* = 3.44).

1. FAR to No-Go stimuli are commission errors to K or alcohol images - when participants are not meant to respond but do. The ‘hit rate’ to Go trials is equivalent to omission errors (proportion of stimuli responded to correctly) [↑](#footnote-ref-1)
